# Research progress on Brassicaceae plants: a bibliometrics analysis

**DOI:** 10.3389/fpls.2024.1285050

**Published:** 2024-01-31

**Authors:** Ruixue Zhou, Xinsheng Qin, Junjun Hou, Yining Liu

**Affiliations:** ^1^ College of Forestry and Landscape Architecture, South China Agricultural University, Guangzhou, China; ^2^ College of Horticultural Science and Technology, Hebei Normal University of Science and Technology, Qinhuangdao, China

**Keywords:** Brassicaceae, visual analysis atlas, evolution, plants, phylogeny

## Abstract

The Brassicaceae is a worldwide family that produces ornamental flowers, edible vegetables, and oilseed plants, with high economic value in agriculture, horticulture, and landscaping. This study used the Web of Science core dataset and the CiteSpace bibliometric tool to quantitatively visualize the number of publications, authors, institutions, and countries of 3139 papers related to Brassicaceae plants from 2002 to 2022. The keywords and references were divided into two phases: Phase 1 (2002-2011) and Phase 2 (2012-2022) for quantitative and qualitative analysis. The results showed: An average annual publication volume of 149 articles, with an overall fluctuating upward trend; the research force was mainly led by Professor Ihsan A. Al-shehbaz from Missouri Botanical Garden; and the United States had the highest number of publications. In the first phase, research focused on the phylogeny of Brassicaceae plants, while the second phase delved into diverse research based on previous studies, research in areas such as polyploidy, molecular technique, physiology, and hyperaccumulator has been extended. Based on this research, we propounded some ideas for future studies on Brassicaceae plants and summarized the research gaps.

## 1 Introduction

The plants of Brassicaceae, characterized by features such as pungent odor (glucosinolates), cruciform corolla, tetradynamous stamens, and siliques, is an important natural family and a globally significant plant family (Zhu et al., 2023). According to the modern angiosperm classification system APG-IV, the Brassicaceae family currently consists of 352 genera and 3350-3660 species (https://duocet.ibiodiversity.net/), and mainly distributed in the northern temperate zone, particularly in the Mediterranean region (Al-Shehbaz, 1984). The Brassicaceae family is highly diverse, comprising two subfamilies: Aethionematoideae and Brassicoideae. In addition, the Brassicoideae are partitioned into five supertribes, including the previously recognized Brassicodae and the newly established Arabodae, Heliophilodae, Hesperodae, and Camelinodae (German et al., 2023). Under the Brassicodae, the Brassiceae are further subdivided into 13 subtribes. Among them, the Brassiceae has the highest number of genera, with 92. Many of these species have important economic value, with *Brassica rapa* var. *oleifera* DC. (rapeseed) being widely cultivated in China as an edible oil crop and ornamental plant. It was worth mentioning that *Arabidopsis thaliana* L., as a model organism, whose genome is the first higher plant genome to be sequenced and it serves as a key comparison point with other eukaryotic genomes, widely used in research areas such as plant genetics (Polanco and Ruiz, 2002), cell biology (Labuz et al., 2010), and molecular biology (Klepikova et al., 2016). For example, *Arabidopsis thaliana*, as one of the earliest model organisms worldwide, has been utilized in several comprehensive studies for enhancing genome annotation, profiling organelles, tissues, cells, or subcellular proteomes, as well as investigating developmental processes and responses to biotic and abiotic stresses through differential relative and absolute quantitative strategies (Wienkoop et al., 2010). Apart from the Brassicaceae family being an important area of study, individual tribes within it are also worth mentioning, especially those that hold significant value, such as the Chorisporeae (German et al., 2011). Due to its diverse species, various types of research have been constantly conducted on. *Arabis alpina* L., a high-altitude plant in the Brassicaceae family, it has become a complementary model to *Arabidopsis thaliana* for studying evolutionary life history traits such as perenniality and ecological genomics in harsh environments (Wötzel et al., 2021).The glucosinolate-myrosinase system, discovered in the order Brassicales, is one of the best-studied plant chemical defense systems. The defensive function of the glucosinolate-myrosinase system has been confirmed in various studies targeting different insect herbivores. However, many versatile and specialized herbivores utilize glucosinolate-containing plants as hosts, leading to significant agronomical losses in crops such as oil seed rape and other Brassicaceae species. Significant progress has been made in understanding how specialized insect herbivores overcome the glucosinolate-myrosinase system and even utilize it for their own defense purposes (Winde and Wittstock, 2011). Many scholars have conducted comprehensive review studies on various aspects of Brassicaceae plants, including phylogenetics (Al-Shehbaz et al., 2006), genomics (Augustine et al., 2013), resistance breeding (Diederichsen et al., 2009), and self-incompatibility (Hiscock and McInnis, 2003). However, these studies are mostly focused on specific perspectives, and the visual bibliometric analysis is still very rare.

Despite a large body of research literature describing various aspects of Brassicaceae plants, there is a lack of integration of effective discipline-specific information due to variations in authors, institutions, publication dates, and journals. Consequently, it has been challenging to identify the development trends in this field (Wallin, 2005). Given the absence of research on bibliometric analysis specifically focused on Brassicaceae plants, this study was of significant importance as it aimed to provide timely insights into the developmental dynamics and future research trends of this plant family.

As the quantity of published literature has exploded in various fields, bibliometric analysis, as a statistical method for reviewing and describing published articles, can reveal the direction of disciplinary development and the dynamics of research from multiple perspectives, aiding researchers in evaluating academic research in a particular field (Rey-Martí et al., 2016). Qualitative analysis in a single field often struggles to capture research frontiers and hot topics. However, by utilizing scientific knowledge graph tools for bibliometric analysis, researchers can quantitatively and objectively understand the research status and focal points of knowledge structures, promoting cross-disciplinary integration and generating new research ideas (Mokhnacheva and Tsvetkova, 2020). CiteSpace, based on various elements in a large number of publications, allows for operations such as keyword clustering analysis, author co-occurrence analysis, and bursting term analysis. Based on these analyses, it generates visual analysis maps that demonstrate the development process and internal connections of a specific discipline, showcasing research trends and hot topics in that field. This enables scholars to explore essential foundational knowledge and research dynamics in their research areas and provides a scientific reference for predicting future development trends through bibliometric analysis. Currently, CiteSpace software is one of the most popular analysis tools in the field of bibliometric analysis and has been widely employed in both social sciences and natural sciences.

## 2 Materials and methods

### 2.1 Data sources

To collect a large amount of data on Brassicaceae plant research topics, we constructed a database of literature on Brassicaceae plant research topics over the past 20 years using the core ensemble of curated literature in the Web of Science (WOS), which is now widely accepted as a database source tool for bibliometric analysis (Chen et al., 2022; Xu et al., 2022). In WOS, we set the subject term as “Brassicaceae” for searching, selected “Science Citation Index Expanded”, and “Social Sciences Citation Index” for the citation index, and selected the type of literature as “Article”. The time of publication was restricted to 2002-2022, and the preliminary search yielded 6273 articles with a search date of July 2023. To exclude disciplines unrelated to Brassicaceae plant research, the WOS categories “Plant Sciences”, “Agriculture”, and “Horticulture” were selected, resulting in a final selection of 3139 papers. These papers were then exported in plain text format in batches for subsequent analysis using CiteSpace software. Finally, a visualization analysis was conducted to generate the corresponding knowledge map. (Relevant data can be found as a zip file in the **Supplementary Material**).

### 2.2 Research methodology

CiteSpace is a visualization and analysis software created by Professor Chaomei Chen, a Chinese-American scholar, which is applied to identify and display new trends and dynamics in the scientific literature (Chen, 2004). The software is free and open source, and the software used in this study was downloaded by the authors from official sources.

The downloaded literature was statistically analyzed by CiteSpace software, and based on the bibliometric statistics, the main research power map and literature co-citation map were constructed; at the same time, the keyword co-occurrence map, hotspot clustering map of keywords, theme evolution map, and keyword mutation map were constructed, so that the hotspots of the research on Brassicaceae and its evolution trend could be analyzed in terms of content.

**Figure 1:**
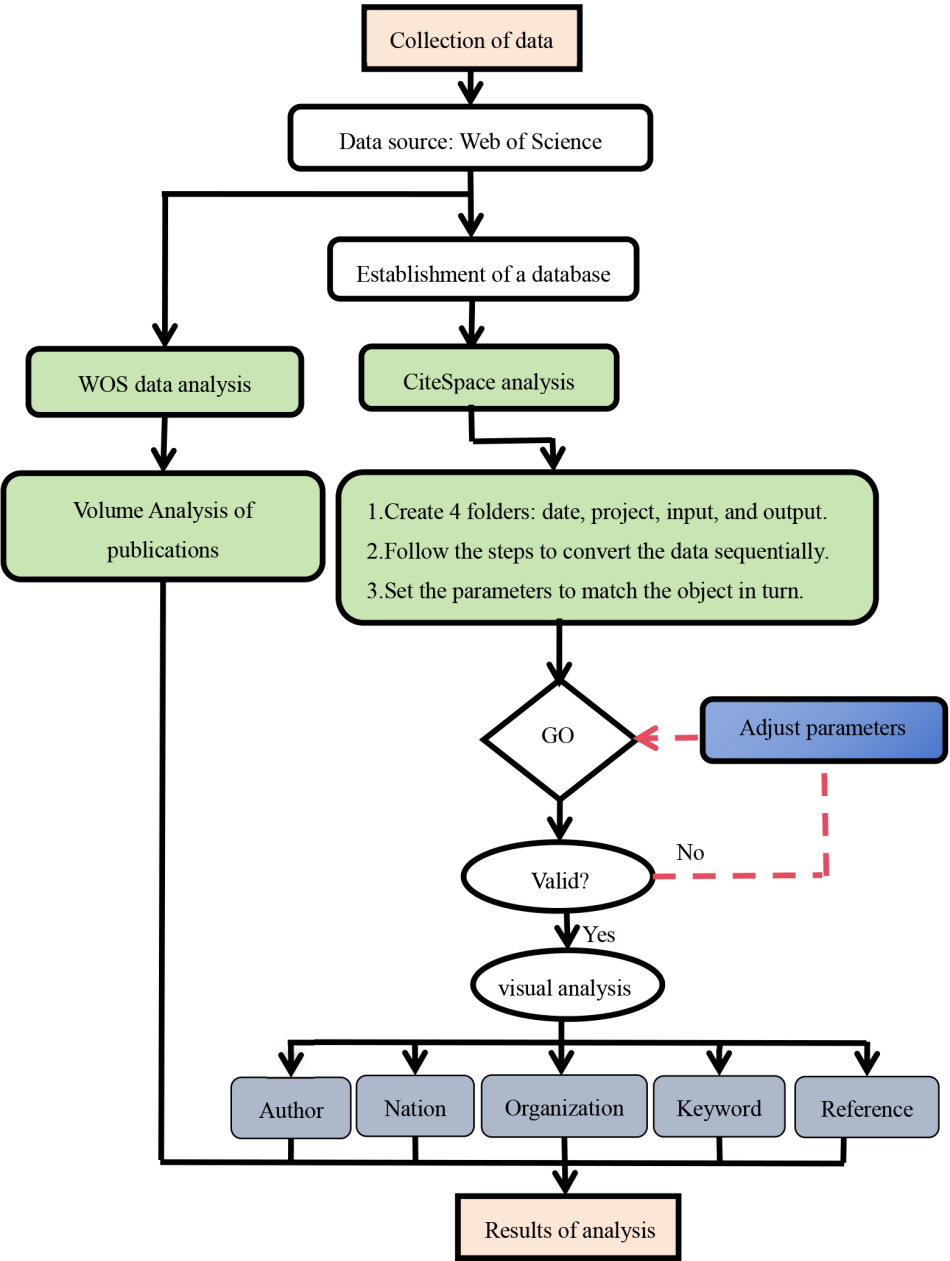
Flowchart of CiteSpace bibliometric analysis.

CiteSpace software parameters were set to the time span for the author, country, and institution was from 2002-2022, while for keyword and reference it was divided into two periods: 2002-2011 and 2012-2022, time slices for Years Per Silce=1. The pruning slice network was selected, with a node threshold of Top N=50, meaning that data extraction was based on the top 50 ranked data for each time slice to generate the final network. The default cosine algorithm was used for the association strength of network nodes, and the k value in the g-index was set to 10. CiteSpace 6.2.R6 basic edition was used for this analysis, and the data results analysis function provided by the WOS was utilized to count the number of publications on Brassicaceae plants. Excel 2016 software was used for chart plotting. Finally, the visualization results were analyzed according to the flowchart steps (**Figure 1**).

## 3 Results and analysis

### 3.1 Analysis of the publication trends

**Figure 2:**
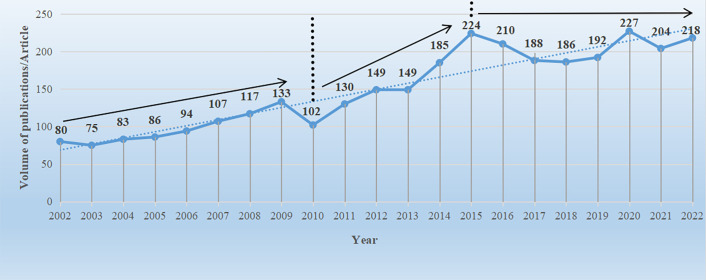
Number of literature issuance volumes for Brassicaceae plant research during 2002-2022. The data in the graph were derived from WOS core journal search results, the solid line represents the direction of literature issuance, and the dashed line indicates the auxiliary trend line.

The analysis of publication data can be visually represented in the form of a line graph, which provides an intuitive reflection of the research development speed and publication quantity in the Brassicaceae family. By utilizing the data analysis functionality of the WOS in conjunction with an Excel spreadsheet, a line graph of the publication quantity in the Brassicaceae family (**Figure 2**) was created. From 2002 to 2022, a total of 3139 published articles related to Brassicaceae plants were identified (Note that the data used in this study was obtained using the filtering method described in section 2.1 and does not represent the entirety of the content in the WOS database). The average annual publication quantity was 149 articles, as a whole, the total publication quantity exhibited a fluctuating upward trend. When examining the data in more detail, it could be roughly divided into three stages: from 2002 to 2010, there was a slow growth phase with an annual growth rate of 2.5% and an average annual publication quantity of 97 articles. There was an upward trend in publication quantity, except for a sudden decline of 31 articles in 2010 compared to the previous year. From 2011 to 2015, there was a rapid growth phase, during which research on Brassicaceae plants flourished. The annual growth rate was 11%, with an average annual publication quantity of 167 articles. 2015 marked the first peak in publication quantity, reaching 224 articles. From 2016 to 2022, there was a steady growth phase, with the highest peak in publication quantity occurring in 2020, reaching 227 articles. By this time, the foundational work had been largely completed, and it was expected that future publication quantity would continue to exhibit a stable growth trend.

### 3.2 Analysis of major research forces

#### 3.2.1 Analysis of authors

**Figure 3:**
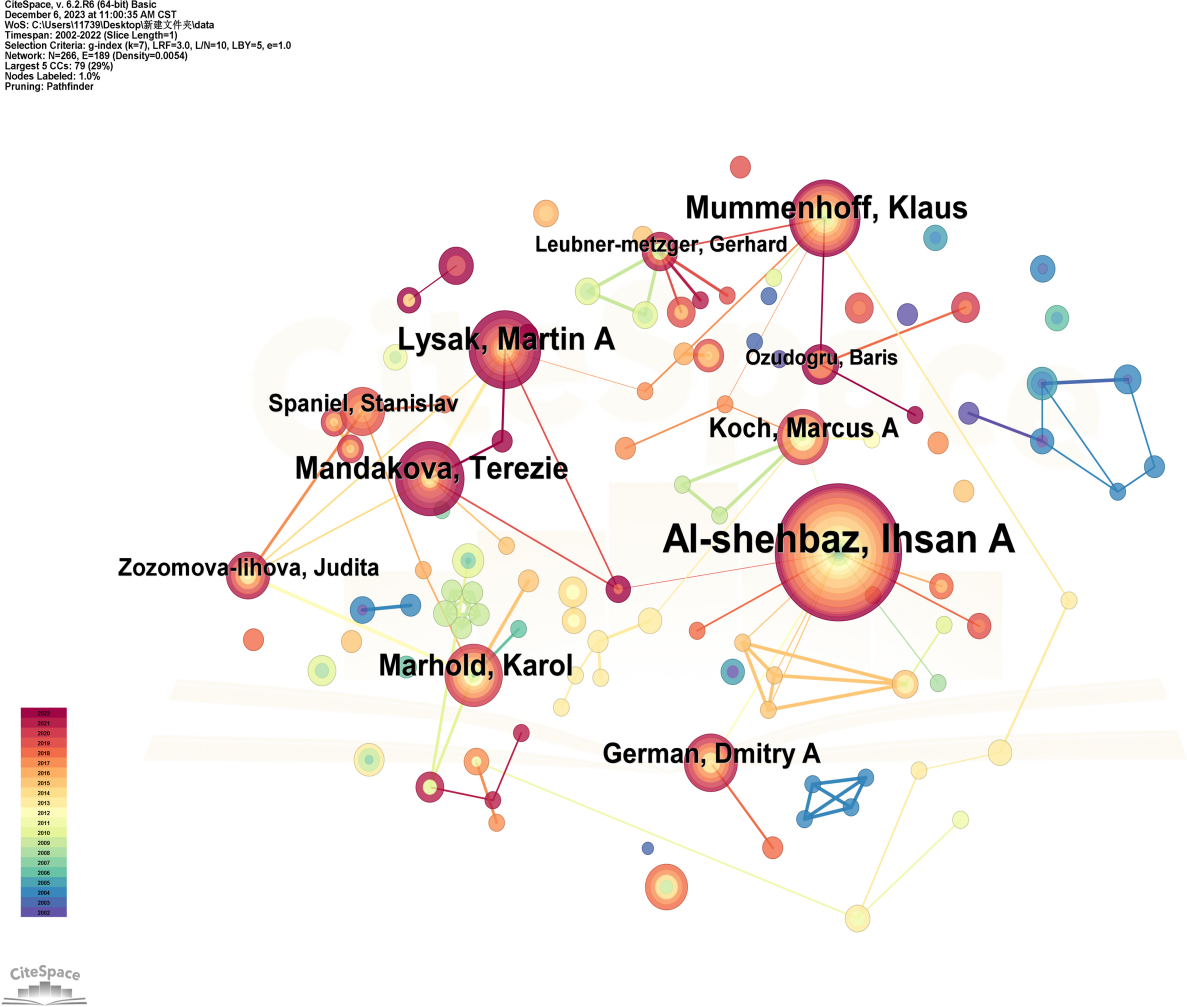
Co-presentation of authors for the studies on Brassicaceae between 2002-2022. The node size represents the amount of the number of issued documents, and the larger the node, the more the number of issued documents of the authors. The network structure reflects the cooperative relationship between authors, the thickness and color depth of the lines represent the intensity of cooperation between authors, and the different colors of the connecting lines represent the different publication times, the brighter the color means that they were published in the last few years, while the darker the color indicates that they were published for a long time.

Research on Brassicaceae plants cannot be separated from related scholars and research organizations, and scholars are the direct producers of articles, which determine the depth and breadth of Brassicaceae plant research. Based on the CiteSpace analysis of the WOS literature data for issuing authors, the literature was generated into an author co-occurrence map (**Figure 3**) as well as the ranking of the amount of issuing articles (**Table 1**). The author co-occurrence graph generated by CiteSpace analysis revealed that the most prolific author in terms of publication quantity between 2002 and 2022 was Al-shehbaz, Ihsan A., with a remarkable total of 116 papers, accounting for 80% of the average annual publication quantity. This signified the long-standing commitment and substantial contribution of this scholar to the study of Brassicaceae plants. The author co-occurrence graph also indicated that in the early years, Al-shehbaz, I.A. had close collaborative relationships with German, Dmitry A., and Koch, Marcus A. However, the intensity of collaboration with other scholars has decreased in recent years, as evident from the varying colors of the connecting lines. In second place was Lysak, Martin A., with a publication count of 41, significantly lower than the top-ranked author. One possible explanation for this difference was the emergence of young researchers who had focused their efforts on conducting a series of studies on Brassicaceae plants, making it more challenging to explore innovative research topics and elevate the level of publication requirements. Additionally, the author co-occurrence graph revealed that Lysak, Martin A. did not have direct collaborative relationships with the top-ranked author, which indirectly underscored the importance of academic exchanges between different fields. Ranking third was Mummenhoff, Klaus, with a publication count of 39. Mummenhoff, K. had collaborative relationships with both the top-ranked and second-ranked authors, with a greater number of connecting lines, forming a localized core author cluster. Although the number of publications was considerably lower than the top-ranked author, Mummenhoff, K. had the highest betweenness centrality score of 0.04 among all nodes, surpassing even the author with the largest node. This indicated that the stronger the collaboration within the primary group of authors, the higher their centrality and influence. In summary, the core group of authors in the early years was centered around Al-shehbaz, I.A., while in recent years, localized networks of author collaboration have been formed around Mummenhoff, K.

**Table 1:** Authors with more than 10 articles for the studies on Brassicaceae between 2002-2022.

Year	Author	Post quantity/Article	Betweenness centrality
2006	Al-shehbaz, Ihsan A.	116	0.03
2013	Lysak, Martin A.	41	0.01
2009	Mummenhoff, Klaus	39	0.04
2013	Mandakova, Terezie	36	0.01
2006	Marhold, Karol	31	0.01
2012	German, Dmitry A.	23	0
2009	Koch, Marcus A.	21	0.03
2012	Zozomova-lihova, Judita	15	0
2016	Spaniel, Stanislav	15	0
2009	Mueller, Caroline	13	0
2009	Leubner-metzger, Gerhard	11	0.01
2018	Ozudogru, Baris	10	0.01

Betweenness centrality is an indicator that evaluates the importance of a node in the whole network, the higher the centrality, the greater its influence in the network.

#### 3.2.2 Analysis of most influential institutions

**Figure 4:**
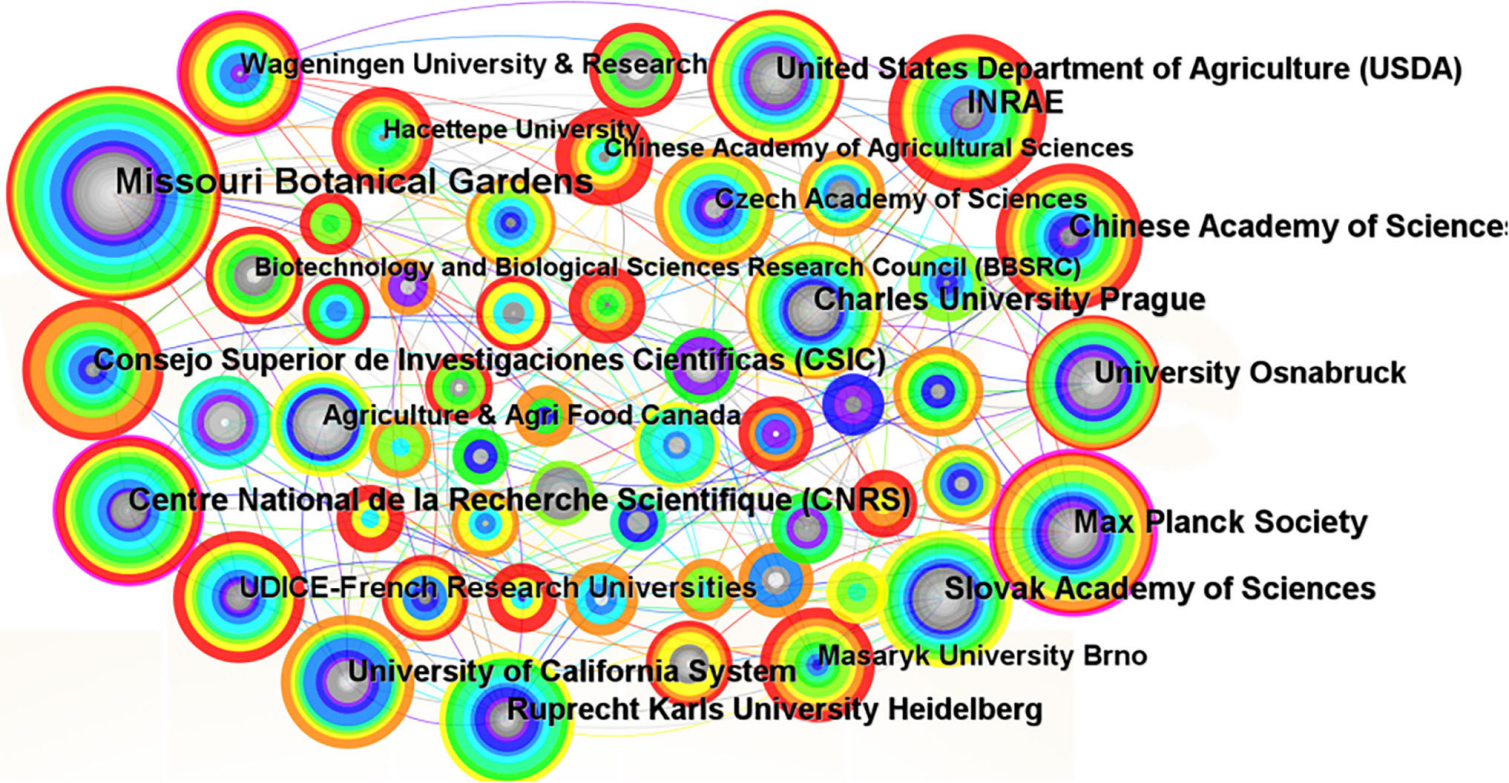
Co-current relationships of institutional cooperation network for the studies on Brassicaceae during 2002-2022.

With the development of scientific research, more and more research is accomplished through cooperation among different countries and institutions, which can generate greater impact and scientific value (Cui et al., 2022). CiteSpace was used to visualize and analyze the documents screened to generate a knowledge graph of the co-occurrence of Brassicaceae plant research institutions (**Figure 4**) and a ranking table of the top 20 issuing institutions (**Supplementary Table 1**), with a total of 208 nodes, 392 line segments, and a network density of 0.0182. Individual institutions were connected in the graph to show their collaborative relationships. The map showed that the lines were dense and concentrated, and there was frequent communication between the major research institutions. The top 20 institutions had a total of 1247 publications, accounting for 40% of the total active literature. The institution with the largest node in the graph was Missouri Botanical Gardens, which was ranked first with 158 publications, accounting for 13% of the total number of publications in the top 20. The second largest node was the Max Planck Society with 85 publications. The purple ring on the outermost layer of the node indicated that the node had a high degree of intermediary centrality, and the larger the node, the higher the centrality and thus the greater the influence. The graph showed that Max Planck Society had a purple ring on the outer ring of the node and the node was larger, with a mediated centrality of 0.23, while Missouri Botanical Gardens, which had the largest number of publications, did not have a purple ring on the outer ring of the node, with a mediated centrality value of only 0.09. This suggested that close communication and cooperation between research organizations could better promote the development of the discipline and more effectively promote the innovative development of the whole Brassicaceae plant research system.

#### 3.2.3 Analysis of countries

**Figure 5:**
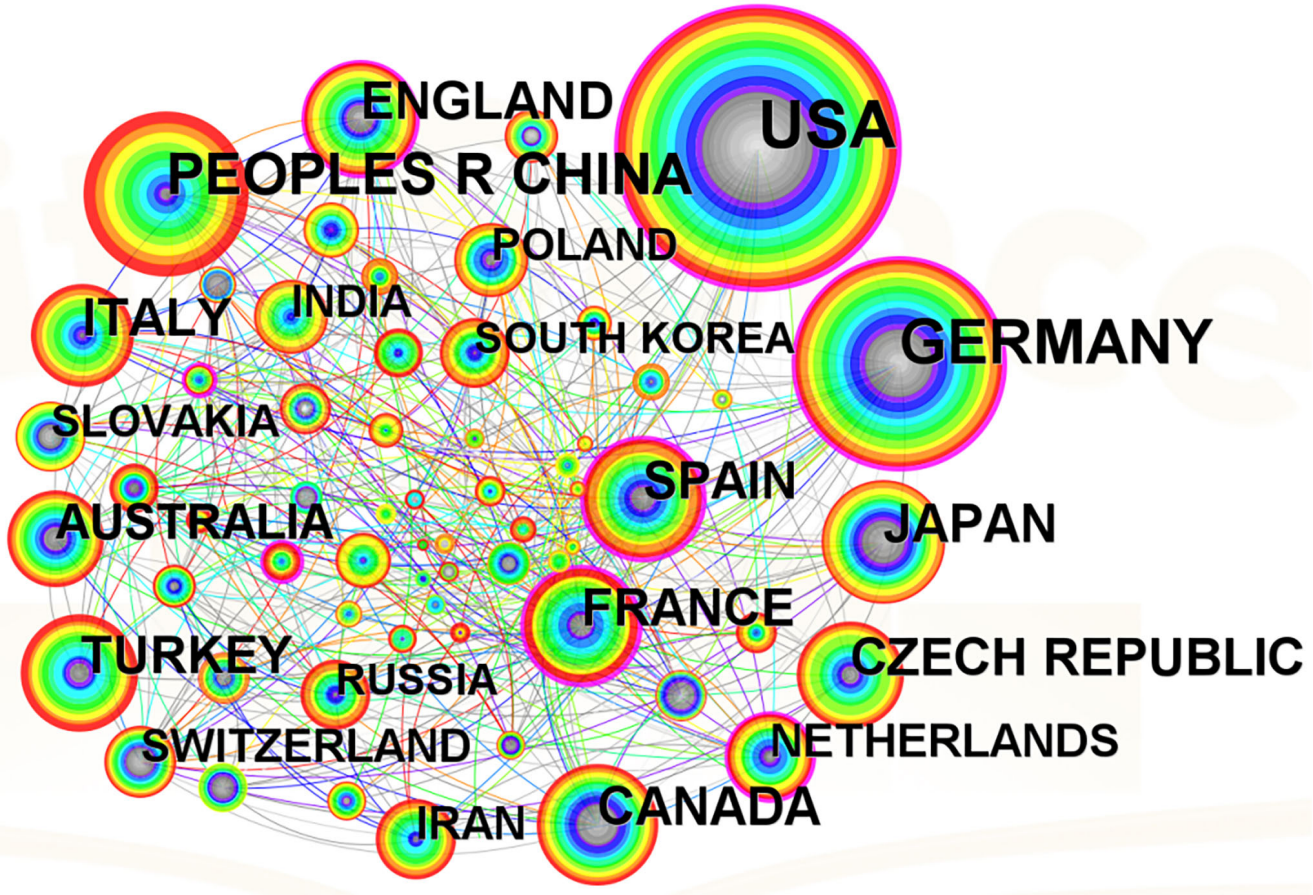
Countries of origin and partnerships on Brassicaceae during 2002-2022.

The number of papers indexed by the Science Citation Index (SCI) to some extent reflects a country’s research strength and influence in a particular research field. Based on CiteSpace analysis of the publications on Brassicaceae plants in the WOS from 2002 to 2022, a network graph was created (**Figure 5**) and a bar chart was used to visually represent the publication counts of different countries (**Supplementary Figure 1**). The network graph and bar chart revealed that the main publishing countries were distributed in Asia, Europe, North America, and Oceania. Among them, the United States had the highest number of publications, with a total of 830 papers, accounting for 27% of the total valid publications. The country with the highest betweenness centrality score was also the United States, with a value of 0.25. Germany and China ranked second and third with 484 and 335 papers respectively. Canada, Japan, Spain, and France followed closely behind with publication counts surpassing 100 papers. The connections in the network graph represented collaborative relationships with other countries, and the more connections there were, the closer collaboration. Additionally, countries with larger nodes and a purple outer ring had a higher betweenness centrality, indicating that the United States and Germany held important positions in the research field of Brassicaceae plants. Since Brassicaceae plants gained significant attention in the scientific community in 2002, the United States has embarked on early research in this area, resulting in the highest number of publications and notable achievements. Canada, Australia, Germany, Italy, the United Kingdom, France, and Japan had also begun research and achieved certain results at an early stage. With the opening-up policy and response to global collaboration, China gradually deepened its research on Brassicaceae plants and actively collaborated with foreign scholars and institutions.

### 3.3 Keyword analysis

Keywords are the essence and focus of each article. They provide a concise summary of the entire article and possess strong generality. The frequency of keywords reflects the frontier hotspots of research topics during a certain period and the core strength of the keywords in the network (Ding and Yang, 2020). Betweenness centrality is a measure of the centrality of a node in a network. The higher the centrality, the greater the influence of the node in the network. When the betweenness centrality of a node is ≥0.1, it is considered a key node. From the perspective of scientometrics, literature with high betweenness centrality values usually plays a significant role in the information transmission process (Qian, 2014). This study focuses on exploring the recent research trends and future directions of Brassicaceae plants by comparing the visualized results of keywords from the periods 2002-2011 and 2012-2022.

#### 3.3.1 Analysis of co-occurrence

**Figure 6:**
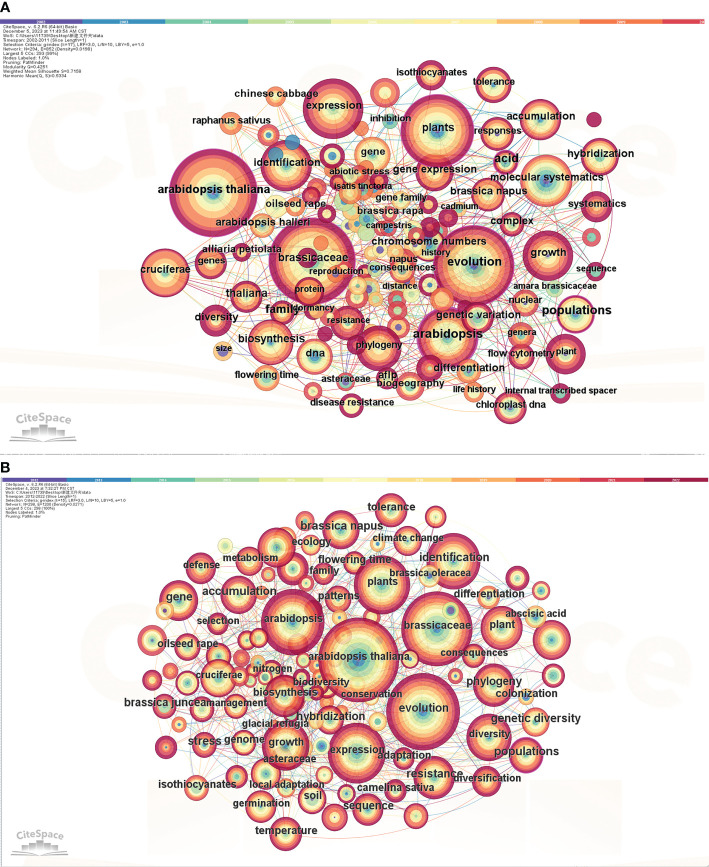
Co-occurrence of keywords for the studies on Brassicaceae during 2002-2011 **(A)** and 2012-2022 **(B)**. The size of nodes represented the frequency of keyword occurrence, with larger nodes indicating higher frequency and representing the research hotspots of the article.

Using CiteSpace, we exported data from the years 2002-2011 and 2012-2022. The node type selected was “Keyword”, and the time slice was set to 1 year. Through pruning the slice network, keyword frequency and betweenness centrality analyses were conducted. Upon running the analysis, we obtained a co-occurrence network graph of keywords (**Figure 6**) and keyword information (**Table 2**). Based on the analysis of the chart, it can be observed that there are only 3 keywords with a frequency of over 100 in phase 1 (2002-2011). However, in phase 2 (2012-2022), there are 9 keywords, which is three times more than the former. The main keywords (phase 1) are *Arabidopsis thaliana*, Brassicaceae, and Evolution. In phase 2, in addition to these three keywords, there are also *Arabidopsis* (DC.) Heynh., Expression, Plants, Identification, Growth, and Diversity. With the progress of science and the development of the era, more profound and novel topics are constantly emerging. CiteSpace can be used to find the most frequently cited literature related to a specific node, For example, the articles (**Figure 6A**) related to the keyword “*Arabidopsis thaliana*” was Brown et al. (2003) analyzed the glucosinolate content of various organs of the model plant *Arabidopsis thaliana*, Columbia (Col-0) ecotype at different stages during its life cycle. They thought that dormant and germinating seeds had the highest concentration, followed by inflorescences, siliques (fruits), leaves, and roots. Rather, during seed germination and leaf senescence, there were significant declines in glucosinolate concentration. This article mainly discusses the physiological and ecological significance of the aforementioned findings, providing valuable insights for future research on plant physiology and biochemistry. Furthermore, the keyword (**Figure 6B**) was also “*Arabidopsis thaliana*”, the most highly cited article was related to the topic of transgenerational increased resistance in plants, Rasmann et al. (2012) designed a series of experiments to investigate the role of jasmonates and siRNA in transgenerational induced resistance to herbivory in two well-studied model species, *Arabidopsis* and tomato (Solanum lycopersicum). Their results showed that both *Arabidopsis* and tomato plants were subjected to herbivory are more resistant to subsequent attacks in the next generation. In summary, *Arabidopsis thaliana* serves as a “treasure trove” for researchers in the scientific community, particularly in the field of plant physiology. In addition, the presence of five keywords in phase 2 that are not present in phase 1, such as Expression and Identification, indicates that research on Brassicaceae plants began in 2012. Specifically, there has been a gradual involvement of *Arabidopsis thaliana* research at the genetic and hereditary level.

**Table 2:** Information on keywords with a posting frequency of more than 100 times in two phases.

Rank	Years			
	2002-2011		2012-2022	
	keyword	times	keyword	times
1	*Arabidopsis thaliana*	144	*Arabidopsis thaliana*	317
2	Brassicaceae	140	Evolution	262
3	Evolution	123	Brassicaceae	247
4	/	/	*Arabidopsis*	203
5	/	/	Expression	176
6	/	/	Plants	159
7	/	/	Identification	143
8	/	/	Growth	118
9	/	/	Diversity	105

Frequency refers to the number of times keywords appear in the retrieved papers, and keywords with higher frequency can to some extent reflect the research hotspots in the field.

#### 3.3.2 Analysis of clustering

**Figure 7:**
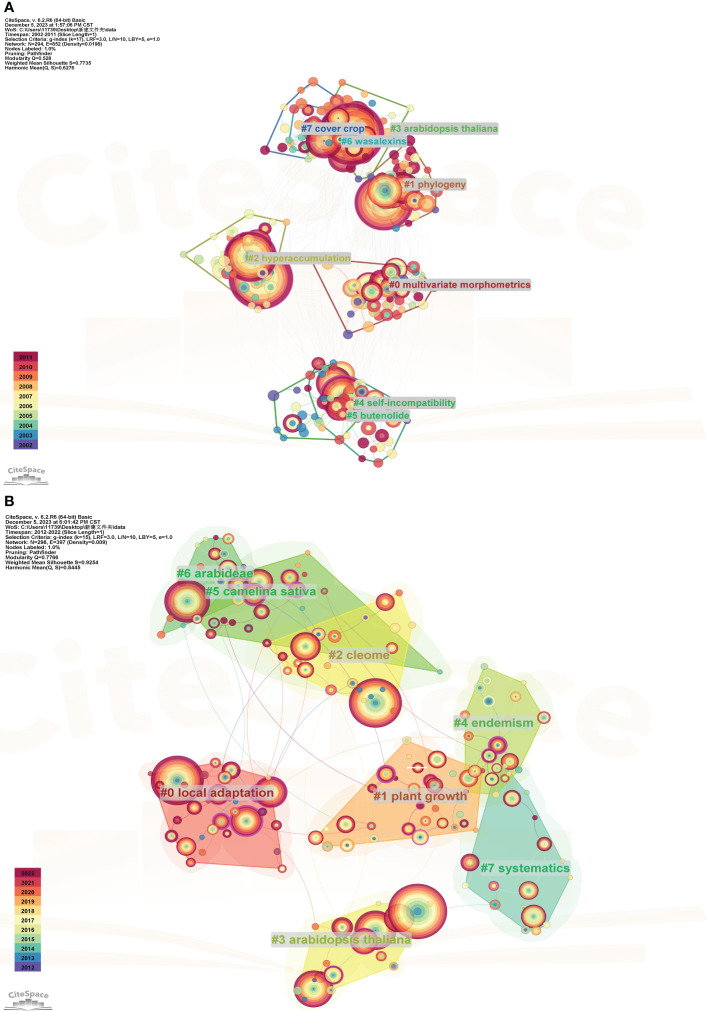
Clustering analysis of keywords for the studies on Brassicaceae during 2002-2011 **(A)** and 2012-2022 **(B)**.

In this study, the clustering labels were extracted using the clustering function and default LLR algorithm in CiteSpace. The software calculated the modularity value (*Q*) and average silhouette value (*S*) based on the network structure and clustering clarity, which were used to assess the visualization effect. Generally, a *Q* value between 0.4-0.8 indicates a reliable network structure. The Silhouette index, which measures the similarity within clusters, typically ranges from 0-1, with higher values indicating higher similarity (Zhu et al., 2018). In the first stage, the *Q* value reached 0.528, and the Mean Silhouette value was calculated as 0.7735, indicating a highly reliable network structure and similarity between clusters. In the second phase, the *Q* value reached 0.7766, and the Mean Silhouette value reached 0.9254, It was proven that the clustering at this stage was highly plausible and suggested that the study on Brassicaceae plants was extensive and characterized by a rich and well-defined range of topics. The different colors of the blocks and cluster labels correspond to different periods. The more vibrant the color, the earlier the research topic, indicating that it is continuously evolving and developing, rather than being from previous years. In **Figure 7A**, the largest cluster label is “multivariate morphometrics”, which contains 34 subclusters. In **Figure 7B**, the largest cluster label is “local adaptation”, with 42 subclusters.

**Table 3** listed the cluster members for each period, highlighting the differences between the two periods. The bolded entries were those that appear in both stages, with “*Arabidopsis thaliana*” appearing four times consecutively, indicating its widespread use as a model organism across various fields. Additionally, the term “taxonomy” in the first stage changes to “phylogeny” in the second stage, demonstrating that research related to Brassicaceae plants had evolved from basic classification work to species evolution and racial development.

**Table 3:** Comparison of keyword clustering information between the two stages.

Cluster-ID	Years					
	2002-2011			2012-2022		
	Sise	Silhouette	LLR	Sise	Silhouette	LLR
#0	34	0.933	Local adaptation; biological invasions; flowering time; **Phenotypic plasticity**; aflp	42	0.764	Multivariate morphometrics; chromosome numbers; flow cytometry; **phenotypic plasticity**; polyploidy
#1	33	0.943	plant growth; biofumigation; **Cover crops**; *sinapis alba*; *brassica juncea*	41	0.742	Phylogeny; its; ***arabidopsis thaliana***; *arabis*; ndhf
#2	23	0.918	Cleome; taxonomy;annotation; turkey; new species	40	0.741	Hyperaccumulation; zinc (zn); phytoremediation; nickel; ***arabidopsis halleri***
#3	20	0.938	***Arabidopsis thaliana***; protein; identification; *brassica rapa*; **expression**	38	0.734	***Arabidopsis thaliana*; brassica napus**; camalexin; *brassica oleracea*; **expression**
#4	20	0.875	Endemism;nickel; ***arabidopsis helleri***; flowers	28	0.796	Self-incompatibility; inflammation; tapetum; resistance; host-pathogen interaction
#5	19	0.94	*Camelina sativa*; *raphanus sativus*; resistance; *brassica rapa*; ***brassica napus***	27	0.836	Butanolide; pollen competition; seed germination; seed dormancy; soil seed bank
#6	19	0.957	Arabideae; ***arabidopsis thaliana***; Miocene; mustards; symbiosis	21	0.836	Wasalexins; **Cruciferae**; condensing enzyme; abiotic stress; methionine
#7	19	0.955	Systematics;**Cruciferae**;vascular flora; pollen; turkey	21	0.727	**Cover crop;** *Alliaria petiolata*; Biofumigation; integrated weed management; widely targeted metabolomics

### 3.4 Reference analysis

The definition of co-citation is when two published articles are cited together by another publication. The citation frequency of a literature can quantify its academic influence. Generally, co-cited articles share similar topics and research backgrounds, and the number of citations can serve as an indicator of their impact level. It is also a way to measure the relationships between different papers and identify core papers that play a key role in the field (Chen, 2012). Consistent with the keyword analysis approach, the entire period is divided into two phases to discuss the clustering of cited literature and the burst of emergent literature.

#### 3.4.1 Cluster analysis of cited literature

**Figure 8:**
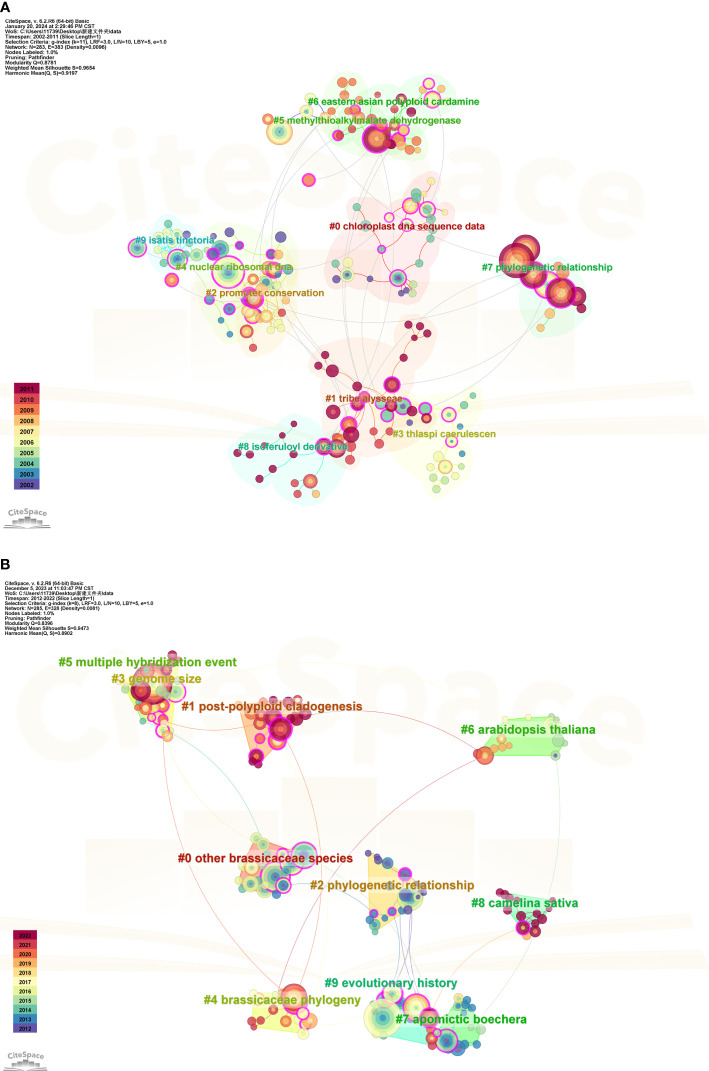
Clustering analysis of cited references for the studies on Brassicaceae during 2002-2011 **(A)** and 2012-2022 **(B)**.

To gain a clearer understanding of the research trends in Brassicaceae plants, we further extracted the co-citation network using CiteSpace. By selecting “Reference” as the node type in the CiteSpace interface, we obtained a cluster map of the cited literature as shown in **Figure 8**. In **Figure 8A**, the *Q* value was 0.8781, and the Mean Silhouette value reached 0.9654, which was close to 1. This indicated that the cited literature within this cluster had a high degree of similarity and close connections to each other. The largest cluster label was “chloroplast DNA sequence data”, consisting of 26 sub-members. The most highly cited article in this cluster was a review by Al-Shehbaz on the systematic and phylogenetic studies of Brassicaceae published in the cluster #7 “phylogenetic relationships” (Al-Shehbaz et al., 2006). They conducted a critical review of the traits used in Brassicaceae plant systematics and discussed the origin, classification, and delineation of genera within this family. In **Figure 8B**, the co-citation cluster had a *Q* value of 0.8396 and a Mean Silhouette value of 0.9473, indicating that the generated cluster structure was reliable and convincing. The most highly cited literature in this cluster, with 122 citations, belonged to cluster #5 “multiple hybridization events”. It referred to an R project supported by the R Core Team, which was widely popular, especially in the field of statistics. Research on Brassicaceae plants often involved physiological, biochemical, molecular genetic, and genetic aspects, requiring extensive database analysis. Therefore, using the R language can effectively assist in data computation and analysis.

#### 3.4.2 Analysis of citation burst

**Figure 9:**
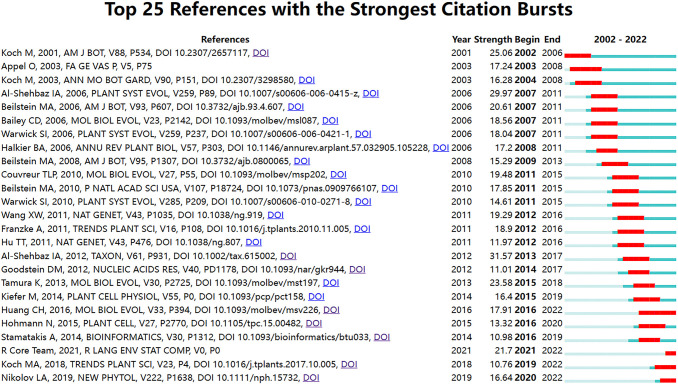
The strength and period of the emergence of 25 subject terms during 2002-2022. The red parts indicate that the references are prominent during this period, while the blue parts are not prominent.

The detection of key term bursts can be used to identify rapidly growing specialized vocabulary in a short period. There are two ways to rank burst literature based on the existing features of CiteSpace (Shi and Liu, 2019): one is by burst start time, and the other is by burst strength. This study adopts the former method for ranking. Based on the temporal distribution and dynamic changes of burst terms, this reflects the research hotspots or new development trends during that period (Chen et al., 2012). An increase in citation bursts indicates that the scientific community is or has been particularly interested in these articles (Su and Lee, 2010). This paper employed CiteSpace to perform a visual analysis and successfully identified highly cited references. The Burstmess function in WOS was used, setting the γ value at 0.8, to reveal 25 burst references (**Figure 9**). The burst intensity analysis no longer compares two stages but rather highlights the popularity of individual publications or a set of publications and predicts future research trends. As shown in the graph, it can be seen that the top two papers in terms of emergence intensity were both authored by Al-Shehbaz. The first one, published in 2012, was titled “A generic and tribal synopsis of the Brassicaceae (Cruciferae)”; the second one, published in 2006, has been analyzed in section 3.4.1. It was evident that Professor Al-Shehbaz was not only an important botanist in the field of Brassicaceae but also his publications were closely related to the taxonomy of Brassicaceae and provided a valuable reference for other scholars. Additionally, based on the duration of burst intensity, the publication by Chinese scholar Huang et al. (2015) remained a burst term for six years, indicating that the articles published by this research group were among the hot topics in the field. They primarily utilized nuclear genes to resolve the phylogeny of the Brassicaceae family, revealing a nested radiation and providing support for convergent morphological evolution.

## 4 Discussion

The family Brassicaceae is a highly diverse and globally distributed group of plants that plays a significant role in the plant kingdom, and their evolutionary history is intricate and fascinating. No studies have been found so far (not retrieved in WOS) regarding bibliometric analysis of Brassicaceae plants. Despite many areas that need improvement, the study has also achieved remarkable results.

### 4.1 Global contribution to Brassicaceae research

The trend in the publication volume of literature to some extent reflects the research intensity in a particular field during a certain period. The research on Brassicaceae plants has been through three stages in terms of publication volume: a slow development stage (2002-2010), a rapid growth stage (2011-2015), and a steady development stage (2016-2022). Overall, there was an upward trend in the publication volume of literature (**Figure 2**), indicating that the research content related to Brassicaceae plants was constantly expanding. Early on, a group of distinguished scientists emerged, including prominent authors such as Al-shehbaz, Koch, and Mummenhoff (**Table 1**). Their collaborative review article published in the Annals of the Missouri Botanical Garden effectively summarized the research achievements in the systematic taxonomy, population genetics, and evolutionary biology of Brassicaceae plants prior to the 20th century. They critically discussed the traditional and molecular-based phylogenies, new generic alignments were proposed, and groups in need of molecular studies were identified(Koch et al., 2003). The final advice given was the future directions of research should move beyond assessing generic relationships or limits, and should address character development and evolution, the molecular basis of various homoplastic characters, the nature of the genome, and many other new challenges that are emerging from detailed molecular studies of *Arabidopsis thaliana*. This finding further confirms the position of the third-ranked article in the burst analysis of cited literature, which had a sustained burst duration of four years. Subsequently, an increasing number of research institutions and countries began to actively engage in Brassicaceae plant research.

Missouri Botanical Gardens is the oldest botanical garden in the United States and is now one of the world’s leading botanical research institutions, with core research on conserving plant diversity. After conducting a literature citation metrics visualization analysis using CiteSpace, we obtained results in the form of network graphs and rankings based on publication frequency. Missouri Botanical Gardens is the most influential and highly-contributing institution. Importantly, the articles published by this institution have provided valuable theoretical guidance and conceptual frameworks for future research on Brassicaceae plants. The institution primarily focuses on research related to the phylogenetics and population relationships of Brassicaceae plants. For example, institutional scholars were based on oil analyses of the ITS (internal transcribed spacer) regions of the nuclear ribosomal DNA and the chloroplast trnL-intron and spacer sequences from 72 American Draba taxa and 6 European Draba species to conduct a systematic analysis. Their analysis discovered that the existing tribal classifications of the Brassicaceae were mostly artificial and that the segregation of Draba and Arabis into separate tribes or subtribes did not accurately reflect their phylogenetic relationship (Koch and Al-Shehbaz, 2002). Based on the above analysis, it is evident that a scholar’s popularity, as reflected in the citation of their publications, can elevate not only their affiliated institution but even their country to a leading position in a particular field or discipline, placing them at the forefront of their time. In terms of national publication volume analysis, the United States holds the top position, followed by Germany and China. This was closely related to the rankings of research institutions and authors based on publication volume, with authors from institutions that published more articles also producing a higher number of publications. This indicated a higher level of emphasis or priority given to the topic by their respective countries (**Supplementary Table 1**, **Table 1**). It was also strongly influenced by the primary distribution areas of Brassicaceae plants (Al-Shehbaz, 1984). The scientific research strength of the United States is widely recognized, and its influence is undeniable.

### 4.2 Advance in Brassicaceae research

The above analysis mainly indicated the quantitative analysis of Brassicaceae, and in the keyword analysis of literature metrology, clustering functions could be used to aggregate related literature or topics together, giving each cluster a specific clustering label. Combining the characteristics and progress of Brassicaceae research, five aspects will be discussed.

#### 4.2.1 Phylogeny of the Brassicaceae

The Brassicaceae family is a plant family of significant economic value and scientific importance, particularly in the fields of agriculture and scientific research. It encompassed several model species and was becoming a model for evolutionary studies at the family level. However, resolving the phylogenetic relationships within the family has been challenging, and there was still a lack of large-scale molecular systematic studies with comprehensive sampling and gene coverage (Couvreur et al., 2009). To our knowledge, several scholars have conducted numerous review studies in this area, including a highly cited review by Al-Shehbaz et al. (2006) on the systematics and phylogeny of the Brassicaceae family. Schranz et al. (2006) summarized advances in their understanding of phylogenetics, polyploidization, and comparative genomics in the family Brassicaceae. At that time, their findings paved the way for a unified comparative genomic framework.

In the first phase of the citation clustering analysis (2002-2011) as shown in **Figure 8A**, it was noteworthy that there were studies focusing on phylogenetic relationships using nuclear ribosomal DNA ITS sequences and chloroplast DNA trnL-F sequences, which also served as cluster label names. As early as 2002, researchers conducted phylogenetic studies on the Alliaria genus within the Brassicaceae family. *Sisymbrium* exemplifies a lack of clear generic boundaries and unique synapomorphies (Al-Shehbaz, 1988). Warwick et al. (2002) Used sequence data from the internal transcribed spacers of nuclear ribosomal DNA and the 5.8S rRNA gene (collectively, ITS region), examined the evolutionary relationships of Old and New World *Sisymbrium* species with its segregate genera and the validity of O.E. Schulz’s classical sectional treatment of *Sisymbrium*. For a long time, the Brassicaceae and Capparaceae families have been considered closely related, with the monophyly of the Capparaceae family being called into question. Hall et al. (2002) analyzed sequence variation for a large sampling, especially of Capparaceae, of these two families, using two chloroplast regions, trnL-trnF and ndhF. Their results elucidate the relationship between Brassicaceae and Capparaceae as well as address infrafamilial relationships within Capparaceae.

The Brassicaceae family has long been known to present significant challenges in the classification and delineation of its genera and tribes. Fortunately, substantial progress has been made in recent years in understanding the phylogenetic relationships and establishing tribal classifications. In the clustering of cited references at the second stage, the labeled theme was presented as “other Brassicaceae species, Phylogenetic relationship, and Brassicaceae phylogeny” (**Figure 8B**), indicating that research on the phylogenetics of the Brassicaceae family had reached a new level. It is well-known that among families with a large number of members, the Brassicaceae family is one of the best-studied in terms of phylogenetics (Warwick et al., 2010; Al-Shehbaz, 2012; Koch et al., 2012). To provide nuclear gene sequence resources for evolutionary research in the Brassicaceae family, Huang et al. (2015) utilized a large dataset consisting of 55 Brassicaceae species to screen a set of marker genes for phylogenetic reconstruction. This approach led to the development of a robust phylogeny, resulting in the first highly resolved framework referred to as Branches A-F, which encompasses six major clades. These results form a foundation for future evolutionary analyses of structures and functions across Brassicaceae. Koch and Grosser (2017) investigated the phylogenetic relationships and evolutionary history of East Asian *Arabis* spp. (Brassicaceae) using plastid trnL-F and nuclear internal transcribed spacer 1 and 2 sequences (ITS). Their study demonstrated that the widespread occurrence of *Arabis hirsute* L. in East Asia, particularly in China, was not supported by their findings. A recent study built upon previous work in the field of Brassicaceae phylogenetics presented the most complete Brassicaceae genus-level family phylogenies to date (Brassicaceae Tree of Life or BrassiToL) based on nuclear and plastome data (Hendriks et al., 2023). Their results strongly supported a recently published new family classification (Classifications mentioned in the Introduction). With a worldwide community of thousands of researchers working on Brassicaceae and its diverse members, this new genus-level family phylogeny will be an indispensable tool for studies on biodiversity and plant biology.

#### 4.2.2 The prominent position of *Arabidopsis thaliana*

*Arabidopsis thaliana* is characterized by its small chromosome number, compact plant size, short life cycle, and abundant seed production. Additionally, it was the first plant to have its genome fully sequenced. The *Arabidopsis* Genome Initiative (AGI), a collaborative effort involving researchers from Europe, Japan, and the United States, took 7 years and cost 70 million USD to complete (Initiative, 2000). From the keywords with a node citation frequency greater than 100 times (**Table 2**), Previous studies focused on the yield of phylogeny; In recent years, the focus of research has expanded to include a broader range of fields, such as gene expression, identification, and genetic diversity at the molecular level. Willing et al. (2015) conducted a *de novo* assembly for the 375 Mb genome of the perennial model plant, *Arabis alpina* L. They emphasized that compared to *Arabidopsis thaliana*, the significant reduction in symmetric CG and CHG methylation indicates a weakened maintenance of DNA methylation in *Arabis alpina*. Whole-genome sequences of members of the Brassicaceae family (including the plant model *Arabidopsis thaliana*) are greatly expanding the scope for comparative genomics among closely related plant species (Wang et al., 2011). Nguyen et al. (2013) built a transcriptome reference from 2047 Sanger ESTs and more than 2 million 454-derived sequence reads, representing genes expressed in developing camelina seeds. These transcriptomic data will be useful for the breeding and engineering of additional camelina seed traits. Fornalé et al. (2014) have characterized *Arabidopsis thaliana* AtMYB7, the closest homolog of AtMYB4 and AtMYB32, described as inhibitors of different branches of phenylpropane metabolism. The results showed that AtMYB7 acts as an inhibitor of flavonol biosynthesis, which led us to propose AtMYB4 and AtMYB7 as part of the regulatory mechanism controlling the balance of major UV-sunscreens in *Arabidopsis thaliana*. A study conducted by Willige et al. (2021) revealed another epigenetic mechanism, indicating that shading effects from neighboring plants can promote increased plant height. The study found that *Arabidopsis* mutants lacking PIFs transcription factors exhibited elongation and rapid growth cessation in simulated shading experiments. More and more scientists were placing greater emphasis on the relationship between plants and the environment, especially in terms of climate (Méndez-Vigo et al., 2019; Pierce et al., 2020). Therefore, in the future, *Arabidopsis thaliana* research at the micro level holds the promise of providing a basis for improving the global environment.

#### 4.2.3 Progress in polyploidy research

The Brassicaceae are well known for taxonomic difficulties mostly caused by the extensive, difficult to interpret, convergent evolution in morphological and anatomical characters and by the limited number of diagnostic characters (Koch et al., 2010). Polyploidization can provide abundant genetic variation for adaptive evolution and species formation. Hohmann et al. (2015)presented a comprehensive time-calibrated framework with important divergence time estimates based on whole-chloroplast sequence data for 29 Brassicaceae species. Their results highlighted polyploidization as an important source for generating new evolutionary lineages adapted to changing environments. The Brassicaceae family, owing to its remarkable species, genetic, and physiological diversity as well as its significant economic potential, has become a model for polyploidy and evolutionary studies. Repetitive polyploidization has played a crucial role in the evolution of the Brassicaceae family (Lysak and Koch, 2011), with nearly half of the Brassicaceae taxa hypothesized to have experienced recent polyploid origins (Franzke et al., 2011). Kagale et al. (2014) by revealing the origins of approximately half of the new/mid-polyploids in the Brassicaceae taxa and identifying coincidences between the diversification of Brassicaceae and significant geological events during Earth’s history in the Neogene period, insights into the evolution of the Brassicaceae have been provided. The origin of certain species in the Brassicaceae family is known to be complex and challenging, such as *Cardamine flexuosa* With. Mandáková et al. (2013) presented a whole-genome cytomolecular map of an allopolyploid plant for the first time, by combining comparative chromosome painting and genomic *in situ* hybridization (CCP/GISH), they elucidate the origin and evolution of the *Cardamine flexuosa* chromosome complement. In conclusion, there is still a long way to go in the research of polyploidy in Brassicaceae plants.

#### 4.2.4 Physiological research

Over the past few decades, there has been a particular focus on edible plants, especially those rich in secondary metabolites. Brassicaceae vegetables are a great source of natural antioxidants, as they have high levels of carotenoids, tocopherols, and ascorbic acid. Strong epidemiological evidence suggests that these compounds may help protect the human body from damage caused by reactive oxygen species (Cartea et al., 2010). In recent years, phenolic compounds have been extensively studied due to their potential health-promoting effects (Vallejo et al., 2002). It was reported that the nutritional interest of *Brassica* L.crops was partly related to their phenolic compound contents. They have been reported to possess many useful properties for human health, the most important action of phenolics was their antioxidant activity (Cushnie and Lamb, 2005). In a study on *Brassica oleracea* L., Podsędek et al. (2006) found that the antioxidant activity between red cabbage and Brussels sprouts was equivalent, being 5 to 2.2 times higher than that of white cabbage and savoy cabbage. Broccoli, as a crop, possesses high antioxidant potential associated with high levels of phenolic compounds (Moreno et al., 2006). In addition to *Brassica oleracea* mentioned above, numerous other Brassicaceae species are rich in phenolic compounds. For example, Bennett et al. (2006) studied the phenolic compound composition in several cruciferous species such as *Diplotaxis erucoides* L., and *Diplotaxis tenuifolia* L.

Plants employ complex defense systems to protect against pests and pathogens, including the production of low molecular weight secondary metabolites with antimicrobial activity. These secondary metabolites are synthesized *de novo* after stress and are collectively referred to as phytoalexins. So far, a total of 44 plant phytoalexins have been isolated from cultivated and Brassicaceae vegetables. The majority of these phytoalexins are sulfur-containing alkaloids synthesized through the biosynthesis of amino acid (*S*)-tryptophan (Ahuja et al., 2012). Detailed studies related to this have been reported (Pedras et al., 2011). Glucosinolates are classic examples of plant compounds that influence insect-plant interactions. They are mainly found in Brassicaceae, which includes several important crops. There are significant quantitative and qualitative differences between plant genotypes, tissues, and developmental stages, which pose specific challenges to herbivorous insects. Although glucosinolates serve as constitutive defenses, their levels are influenced by both abiotic and biotic factors, including insect herbivory (Hopkins et al., 2009). In order to characterize the long-term persistence of plant immunity, a study conducted by Rasmann et al. (2012) examined the plant resistance of *Arabidopsis thaliana* and *Solanum lycopersicum* L. by challenging them with caterpillar herbivory, application of methyl jasmonate, or mechanical damage during the nutritional growth process. The resistance of the offspring was evaluated to assess plant resistance. The observed genetic resistance in Brassicaceae and Solanaceae suggested that this trait may be more widely distributed in plants. Therefore, the phenotypic plasticity mechanism of herbivore-induced epigenetic resistance represented a means to enhance defensive capabilities across generations.

#### 4.2.5 Hyperaccumulator research

In the visualization graph of keyword cluster analysis, the term “Hyperaccumulator” appeared in the second phase(2012-2022), and the cluster encompassed a substantial number of subsets.This suggested that studies related to hyperaccumulators were gradually gaining attention from researchers (**Table 3**). The hazards of heavy metal-contaminated soils to human and animal health are increasingly severe. Efforts to use plants for phytoremediation, the process of removing specific metals from soil through plant uptake, have emerged in the past two decades. Metal-accumulating species can be utilized for either phytoextraction or phytomining (Amabogha et al., 2023; Prasad and De Oliveira Freitas, 2003). Hyperaccumulating plants refer to functional plant groups capable of rapidly and heavily accumulating one or multiple heavy metal elements while maintaining normal growth, reproduction, and resistance to toxicity (Rascio and Navari-Izzo, 2011). Among them, *Thlaspi arvense* L. in the Brassicaceae family can accumulate large amounts of heavy metals such as zinc and cadmium in its aboveground tissues. It is widely recognized as a hyperaccumulator plant. However, due to its small biomass, it is unsuitable for direct use in phytoremediation of heavy metal-contaminated soils. Instead, it is commonly used as a model plant for studying the mechanisms of metal accumulation (Liu et al., 2010). Among the known zinc hyperaccumulating plants, the majority belong to the Brassicaceae family, representing at least three independent evolutionary events of zinc hyperaccumulation (Broadley et al., 2007). *Brassica juncea* (L.) Czern. was an important vegetable-use and oil-use crop worldwide and exhibited the potential to tolerate and accumulate Cd, and it could be considered as a Cd hyperaccumulator plant (Li et al., 2023).Identifying which metal hyperaccumulating plants were safe to consume was crucial to avoid potential health risks. As the Brassicaceae family includes various common crops, it was important to understand the reasons behind significant differences in leaf metal concentrations observed in the field and within populations to better comprehend the phenomenon of metal hyperaccumulation. In the future, further exploration of plants with hyperaccumulation abilities, combined with synergistic interactions of organic defense compounds, can enhance the plants’ inherent defense mechanisms, thereby reducing the use of chemical pesticides.

### 4.3 Tendency in Brassicaceae research

After understanding the past and current situation of research on Brassicaceae plants, it is necessary to explore future research trends, especially for those species threatened or endemic (Perrino and Wagensommer, 2022). The citing literature is the frontier of the research and the cited literature is the theoretical basis of the research. Combining the citation clustering and burst intensity of the cited literature (**Figures 8**, **9**), each stage has its emphasis on research directions, with the second stage being particularly pronounced. In the first stage, the top 10 clusters were analyzed, and the red cluster (the largest cluster) was mainly related to DNA sequences, especially the research on plant phylogeny using nuclear ribosomal DNA ITS sequences and chloroplast DNA trnL-F sequences. The largest cluster in the second stage was related to whole-genome duplications (WGDs; or polyploidization). In addition to Brassicaceae systematics research, other researchers have also investigated plant biogeography and floristic analysis, such as Koch et al. (2010) using cpDNA, found five North American lineages of *Arabis* with distinct distribution patterns, of which only the purple/red-flowered lineage consists of proven diploids that evolved directly from East Asian progenitors. They also provided the first evidence for the systematic circumscription of East Asian *Arabis* taxa, which together with the North American taxa, form one clade distantly related to European and Eurasian *Arabis hirsute*.

In summary, the early research trends and frontiers primarily focused on the phylogenetics of Brassicaceae plants, while in later stages, molecular and genetic approaches were gradually integrated into the systematic foundation to explore the origins and explain the diversity of the extensive Brassicaceae family. In the cluster analysis of cited literature in the second stage, researchers showed interest in the model organism *Arabidopsis thaliana*. The Brassicaceae include several major crop plants and numerous important model species in comparative evolutionary research such as *Arabidopsis*, *Brassica*, *Boechera*, *Thellungiella*, and *Arabis* species. Hohmann et al. (2015) proposed a comprehensive time-calibrated framework based on complete chloroplast sequence data from 29 Brassicaceae species, which provided important estimates of divergence times. Whole-genome duplication (WGD) is a ubiquitous genetic mechanism throughout the history of angiosperms (Soltis et al., 2009). Huang et al. (2020) established a time-calibrated chronogram based on whole plastid genomes comprising representative Brassicaceae taxa and published data spanning the entire Rosidae clade. They also utilized BAMM to investigate the diversity patterns of these tribal-level chronograms and compared them with genome size variation and species richness data from the entire family. Finally, they thought the combined effect of tribal crown group age and net diversification rate (speciation minus extinction) was likely to explain sufficiently species richness across Brassicaceae tribes. In the previous section (3.4.1), it had been analyzed that a popular research focus in recent years is the R language technology supported by the R Core Team. Additionally, there are other relatively novel and long-standing topics concerning oilseed crops. *Camelina sativa* is an annual oilseed crop within the Brassicaceae family, as a climate-resilient oilseed, seed meal, and biofuel (biodiesel and renewable or green diesel) crop received increased attention. Neupane et al. (2022) focused on the study of Camelina sativa and summarized the major biotic constraints limiting its production. Additionally, they provided an overview of strategies to enhance this important and versatile crop in the face of rapidly changing climates, including molecular breeding, rhizosphere microbial communities, genetic engineering, and genome editing methods, aiming to increase its global yield. Brassicaceae plants are predominantly crops, and an important future research direction is how to effectively improve crop yield prediction. Of course, the research on Brassicaceae plants goes far beyond what has been described above. However, the information provided earlier is based on the citation literature generated by the analysis tool of the software used. Due to space limitations, it is not possible to cover all aspects in detail. The specific data analysis of clustered cited literature will be presented in the **Supplementary Materials** (**Supplementary Table 2**).

## 5 Conclusion

Citation analysis using CiteSpace software generated a network graph that visualizes the research landscape. This graph illustrates the citation relationships, co-authorship relationships, and other relevant indicators among different publications, helping us understand the structure and development trends of the research field. This study focused on analyzing the relevant literature from the WOS core dataset, covering the years 2002-2022. CitesSpace software was used to quantitatively visualize the publication count, authors, countries, and research institutions. In terms of qualitative analysis, the study examined the keywords, co-citation analysis, clustering analysis, and burst analysis of the relevant literature from two time periods: 2002-2011 and 2012-2022. These analyses were conducted to map the research topics related to the family Brassicaceae and generate network diagrams.

Overall, the number of articles related to Brassicaceae plants published in WOS has shown an increasing trend. However, as more emerging disciplines and specialties emerge, there will be fewer individuals focusing on Brassicaceae plant research. Currently, the main sources of articles are the Missouri Botanical Garden in the United States, the Max Planck Society in Germany, and the Chinese Academy of Sciences, which are the most active publishing institutions in this field. Comparing keywords and co-cited literature from two periods, the research focus in the first stage was relatively concentrated, with a majority of studies focused on phylogenetics. This was likely because, during the initial stages, researchers primarily laid the groundwork by establishing the fundamental theoretical framework, such as the popular application of the model organism *Arabidopsis thaliana*, understanding the racial relationships, classification methods, and principles of family lineage construction within Brassicaceae. In the later stage, building upon the previous work and theoretical foundations, researchers were able to reach higher levels of analysis by utilizing nucleotide sequences through molecular marker technologies to explain the origin of Brassicaceae biodiversity and establish a universally recognized genomic database for Brassicaceae plants, thereby enhancing the efficiency of future research in molecular directions.

## 6 Strengths and limitations

This study had certain limitations. Compared to review articles, bibliometric analysis can handle thousands or even tens of thousands of publications, and there was no specific and detailed review. However, since the analysis methods are based on publications and their references, they are inevitably influenced by various biases, such as publication bias and citation bias (Urlings et al., 2021; Wu et al., 2023). In addition, there were some limitations of the CiteSpace software when analyzing networks. Due to the large amount of data, not every node will be computed, and only representative and prominent nodes will be presented. Nevertheless, WOS has been widely accepted by researchers and is considered a common tool for retrieval and bibliometric analysis due to its high-quality and comprehensive data (Ding and Yang, 2020). The study served as a summary of the past twenty years of research on Brassicaceae plants. Its purpose was to assist researchers interested in the field of Brassicaceae plants in quickly understanding the current research landscape and hot topics, providing a general direction for future investigations, and identifying areas that require further exploration or updating. In fact, there was a wealth of research content related to Brassicaceae plants, and using “Brassicaceae” as a subject terms had certain limitations. For instance, the purpose of this study was to understand various types of research related to Brassicaceae plants, encompassing both macro aspects such as *Arabidopsis thaliana* and *Brassica* species, as well as micro aspects including phylogenetics, evolutionary biology, molecular breeding, and genomics. The function of CiteSpace software was to extract the desired information from a large amount of tedious and repetitive data. The conclusions drawn from this study covered numerous aspects in both the micro and macro domains. However, it was inevitable that important literature could be missed due to the lack of precision and specificity of the subject terms. This is an area where improvements can be made in similar studies and should be considered for future research endeavors. Through the analysis process, it became apparent that “*Arabidopsis thaliana*” had a very high frequency of occurrence, and there was abundant content related to this species. In the future, researchers can conduct searches using the keyword “*Arabidopsis thaliana*” and combine it with other bibliometric software, such as VOSviewer, to achieve better results. Overall, this study provided a systematic exploration of the development, trends, and prospects of Brassicaceae plant research, deepening our understanding of Brassicaceae phylogenetics and providing a basic understanding of current research dynamics.

## Data availability statement

The original contributions presented in the study are included in the article/**Supplementary Material**. Further inquiries can be directed to the corresponding author.

## Author contributions

RZ: Data curation, Writing – original draft, Writing – review & editing. XQ: Funding acquisition, Writing – review & editing. JH: Data curation, Writing – review & editing. YL: Writing – review & editing.
