# Supplementary material for: Research progress on Brassicaceae plants: a bibliometrics analysis
Source: Front Plant Sci. 2024 Jan 31;15:1285050. doi: 10.3389/fpls.2024.1285050 (PMC10864531; doi:10.3389/fpls.2024.1285050)
Supplement: Supplementary file 2 [file DataSheet_2.docx]

Supplementary Materials

Supplementary Table 1. Top 20 institutions for Brassicaceae research publications between 2002-2022.

| **Rank** | **Institution** | **Post quantity**  **/Article** | **Earliest publication**  **/Year** | **Betweenness centrality** |
| --- | --- | --- | --- | --- |
| 1 | Missouri Botanical Gardens | 158 | 2002 | 0.09 |
| 2 | Max Planck Society | 85 | 2003 | 0.23 |
| 3 | Inrae | 82 | 2007 | 0.09 |
| 4 | Chinese Academy of Sciences | 72 | 2004 | 0.06 |
| 4 | Centre National de la Recherche Scientifique (CNRS) | 72 | 2003 | 0.12 |
| 6 | Charles University Prague | 71 | 2002 | 0.04 |
| 7 | Slovak Academy of Sciences | 68 | 2002 | 0.02 |
| 8 | Consejo Superior de Investigaciones Cientificas (CSIC) | 66 | 2002 | 0.07 |
| 9 | United States Department of Agriculture (USDA) | 65 | 2003 | 0.06 |
| 10 | University Osnabruck | 62 | 2002 | 0.07 |
| 11 | Ruprecht Karls University Heidelberg | 61 | 2004 | 0.05 |
| 12 | University of California System | 60 | 2002 | 0.05 |
| 13 | UDICE-French Research University | 55 | 2004 | 0.06 |
| 14 | Masaryk University Brno | 47 | 2006 | 0.03 |
| 15 | Czech Academy of Sciences | 44 | 2004 | 0 |
| 15 | Wageningen University & Research | 44 | 2006 | 0.14 |
| 17 | Agriculture & Agriculture Food Canada | 39 | 2002 | 0.02 |
| 18 | Hacettepe University | 33 | 2012 | 0.04 |
| 19 | Biotechnology and Biological Sciences Research Council (BBSRC) | 32 | 2002 | 0.01 |
| 20 | Chinese Academy of Agricultural Sciences | 31 | 2011 | 0.03 |

Supplementary Table 2. Reference clustering information table (the table shows the citing articles).

| **Cluster** | **Coverage** | **GCS** | **bibliography** |
| --- | --- | --- | --- |
| **#**0 | 13 | 48 | Lihova, J (2004.0-JAN) [Origin of the disjunct tetraploid cardamine amporitana (brassicaceae) assessed with nuclear and chloroplast dna sequence data](http://dx.doi.org/10.3732/ajb.91.8.1231). AMERICAN JOURNAL OF BOTANY, V91, P12 DOI 10.3732/ajb.91.8.1231 |
|  | 9 | 38 | Marhold, K (2004.0-JAN) [Comparative its and aflp analysis of diploid cardamine (brassicaceae) taxa from closely related polyploid complexes](http://dx.doi.org/10.1093/aob/mch073). ANNALS OF BOTANY, V93, P14 DOI 10.1093/aob/mch073 |
|  | 9 | 40 | Bleeker, W (2002.0-JAN) [Chloroplast dna variation and biogeography in the genus rorippa scop. (brassicaceae)](http://dx.doi.org/10.1055/s-2002-20442). PLANT BIOLOGY DOI 10.1055/s-2002-20442 |
| **#**1 | 18 | 37 | Koch, MA (2010.0-JAN) [Colonizing the american continent: systematics of the genus arabis in north america (brassicaceae)](http://dx.doi.org/10.3732/ajb.0900366). AMERICAN JOURNAL OF BOTANY, V97, P18 DOI 10.3732/ajb.0900366 |
|  | 18 | 144 | Warwick, SI (2010.0-JAN) [Closing the gaps: phylogenetic relationships in the brassicaceae based on dna sequence data of nuclear ribosomal its region](http://dx.doi.org/10.1007/s00606-010-0271-8). PLANT SYSTEMATICS AND EVOLUTION, V285, P24 DOI 10.1007/s00606-010-0271-8 |
|  | 15 | 17 | German, DA (2011.0-JAN) [Molecular phylogeny and systematics of the tribe chorisporeae (brassicaceae)](http://dx.doi.org/10.1007/s00606-011-0452-0). PLANT SYSTEMATICS AND EVOLUTION, V294, P22 DOI 10.1007/s00606-011-0452-0 |

Supplementary Table 2 (continue)

| **Cluster** | **Coverage** | **GCS** | **bibliography** |
| --- | --- | --- | --- |
| **#**2 | 13 | 30 | Oyama, RK (2008.0-JAN) [The shrunken genome of arabidopsis thaliana](http://dx.doi.org/10.1007/s00606-008-0017-z). PLANT SYSTEMATICS AND EVOLUTION, V273, P15 DOI 10.1007/s00606-008-0017-z |
|  | 12 | 38 | Koch, MA (2008.0-JAN) [Arabidopsis thaliana's wild relatives: an updated overview on systematics, taxonomy and evolution](http://dx.doi.org/10.1002/tax.573021). TAXON, V57, P11 DOI 10.1002/tax.573021 |
|  | 11 | 178 | Mandakova, T (2008.0-JAN) [Chromosomal phylogeny and karyotype evolution in x=7 crucifer species (brassicaceae)](http://dx.doi.org/10.1105/tpc.108.062166). PLANT CELL, V20, P12 DOI 10.1105/tpc.108.062166 |
| **#**3 | 7 | 34 | Warwick, SI (2004.0-JAN) [Phylogeny of braya and neotorularia (brassicaceae) based on nuclear ribosomal internal transcribed spacer and chloroplast trnl intron sequences](http://dx.doi.org/10.1139/B04-012). CANADIAN JOURNAL OF BOTANY-REVUE CANADIENNE DE BOTANIQUE, V82, P17 DOI 10.1139/B04-012 |
|  | 6 | 52 | Koch, M (2004.0-JAN) [Taxonomic and phylogenetic evaluation of the american "thlaspi" species: identity and relationship to the eurasian genus noccaea (brassicaceae)](http://dx.doi.org/10.1600/036364404774195566). SYSTEMATIC BOTANY, V29, P10 DOI 10.1600/036364404774195566 |
|  | 5 | 40 | Taylor, SI (2006.0-JAN) [Within and between population variation for zinc and nickel accumulation in two species of thlaspi (brassicaceae)](http://dx.doi.org/10.1111/j.1469-8137.2005.01625.x). NEW PHYTOLOGIST DOI 10.1111/j.1469-8137.2005.01625.x |
| **#**4 | 8 | 59 | Heenan, PB (2002.0-JAN) Molecular systematics of the new zealand pachycladon (brassicaceae) complex: generic circumscription and relationship to arabidopsis sens. lat. and arabis sens. lat.. NEW ZEALAND JOURNAL OF BOTANY, V40, P20 |
|  | 7 | 56 | Warwick, SI (2002.0-JAN) [Phylogeny of sisymbrium (brassicaceae) based on its sequences of nuclear ribosomal dna](http://dx.doi.org/10.1139/B02-089). CANADIAN JOURNAL OF BOTANY-REVUE CANADIENNE DE BOTANIQUE, V80, P16 DOI 10.1139/B02-089 |
|  | 7 | 62 | O'kane, SL (2003.0-JAN) [Phylogenetic position and generic limits of arabidopsis (brassicaceae) based on sequences of nuclear ribosomal dna](http://dx.doi.org/10.2307/3298545). ANNALS OF THE MISSOURI BOTANICAL GARDEN, V90, P10 DOI 10.2307/3298545 |
| **#**5 | 9 | 76 | Sawada, Y (2009.0-JAN) [Omics-based approaches to methionine side chain elongation in arabidopsis: characterization of the genes encoding methylthioalkylmalate isomerase and methylthioalkylmalate dehydrogenase](http://dx.doi.org/10.1093/pcp/pcp079). PLANT AND CELL PHYSIOLOGY, V50, P10 DOI 10.1093/pcp/pcp079 |
|  | 7 | 30 | Ludwig-mueller, J (2009.0-JAN) [Glucosinolates and the clubroot disease: defense compounds or auxin precursors?](http://dx.doi.org/10.1007/s11101-008-9096-2). PHYTOCHEMISTRY REVIEWS DOI 10.1007/s11101-008-9096-2 |
|  | 7 | 69 | Sawada, Y (2009.0-JAN) [Arabidopsis bile acid:sodium symporter family protein 5 is involved in methionine-derived glucosinolate biosynthesis](http://dx.doi.org/10.1093/pcp/pcp110). PLANT AND CELL PHYSIOLOGY DOI 10.1093/pcp/pcp110 |
| **#**6 | 7 | 49 | Marhold, K (2010.0-JAN) [Cytotype diversity and genome size variation in eastern asian polyploid cardamine (brassicaceae) species](http://dx.doi.org/10.1093/aob/mcp282). ANNALS OF BOTANY, V105, P16 DOI 10.1093/aob/mcp282 |
|  | 7 | 17 | German, DA (2011.0-JAN) [Molecular phylogeny and systematics of the tribe chorisporeae (brassicaceae)](http://dx.doi.org/10.1007/s00606-011-0452-0). PLANT SYSTEMATICS AND EVOLUTION, V294, P22 DOI 10.1007/s00606-011-0452-0 |
|  | 7 | 35 | Jordon-thaden, I (2008.0-JAN) [Species richness and polyploid patterns in the genus draba (brassicaceae): a first global perspective](http://dx.doi.org/10.1080/17550870802349112). PLANT ECOLOGY & DIVERSITY DOI 10.1080/17550870802349112 |
| **#**7 | 8 | 39 | Warwick, SI (2009.0-JAN) [Phylogenetic relationships in the tribes schizopetaleae and thelypodieae (brassicaceae) based on nuclear ribosomal its region and plastid ndhf dna sequences](http://dx.doi.org/10.1139/B09-051). BOTANY, V87, P25 DOI 10.1139/B09-051 |
|  | 7 | 38 | Koch, MA (2008.0-JAN) [Arabidopsis thaliana's wild relatives: an updated overview on systematics, taxonomy and evolution](http://dx.doi.org/10.1002/tax.573021). TAXON, V57, P11 DOI 10.1002/tax.573021 |
|  | 6 | 144 | Warwick, SI (2010.0-JAN) [Closing the gaps: phylogenetic relationships in the brassicaceae based on dna sequence data of nuclear ribosomal its region](http://dx.doi.org/10.1007/s00606-010-0271-8). PLANT SYSTEMATICS AND EVOLUTION, V285, P24 DOI 10.1007/s00606-010-0271-8 |

Supplementary Table 2 (continue)

| **Cluster** | **Coverage** | **GCS** | **bibliography** |  |
| --- | --- | --- | --- | --- |
| **#**8 | 8 | 170 | Agerbirk, N (2009.0-JAN) [Indole glucosinolate breakdown and its biological effects](http://dx.doi.org/10.1007/s11101-008-9098-0). PHYTOCHEMISTRY REVIEWS DOI 10.1007/s11101-008-9098-0 |  |
|  | 5 | 32 | Agerbirk, N (2011.0-JAN) [Isoferuloyl derivatives of five seed glucosinolates in the crucifer genus barbarea](http://dx.doi.org/10.1016/j.phytochem.2011.01.034). PHYTOCHEMISTRY, V72, P14 DOI 10.1016/j.phytochem.2011.01.034 |  |
|  | 4 | 16 | Ahuja, I (2011.0-JAN) [Oilseed rape seeds with ablated defence cells of the glucosinolate-myrosinase system. production and characteristics of double haploid mineless plants of brassica napus l.](http://dx.doi.org/10.1093/jxb/err195). JOURNAL OF EXPERIMENTAL BOTANY, V62, P19 DOI 10.1093/jxb/err195 |  |
| **#**9 | 7 | 16 | Condurso, C (2006.0-JAN) [The leaf volatile constituents of isatis tinctoria by solid-phase microextraction and gas chromatography/mass spectrometry](http://dx.doi.org/10.1055/s-2006-946679). PLANTA MEDICA DOI 10.1055/s-2006-946679 | |
|  | 6 | 77 | Recio, M (2006.0-JAN) [Anti-inflammatory and antiallergic activity in vivo of lipophilic isatis tinctoria extracts and tryptanthrin](http://dx.doi.org/10.1055/s-2006-931562). PLANTA MEDICA DOI 10.1055/s-2006-931562 | |
|  | 5 | 93 | Textor, S (2004.0-JAN) [Biosynthesis of methionine-derived glucosinolates in arabidopsis thaliana: recombinant expression and characterization of methylthioalkylmalate synthase, the condensing enzyme of the chain-elongation cycle](http://dx.doi.org/10.1007/s00425-003-1184-3). PLANTA, V218, P10 DOI 10.1007/s00425-003-1184-3 | |

The coverage in the table represents the number of nodes covered by this literature; The GCS in the table represents the total number of times the literature has been cited in the WOS database.


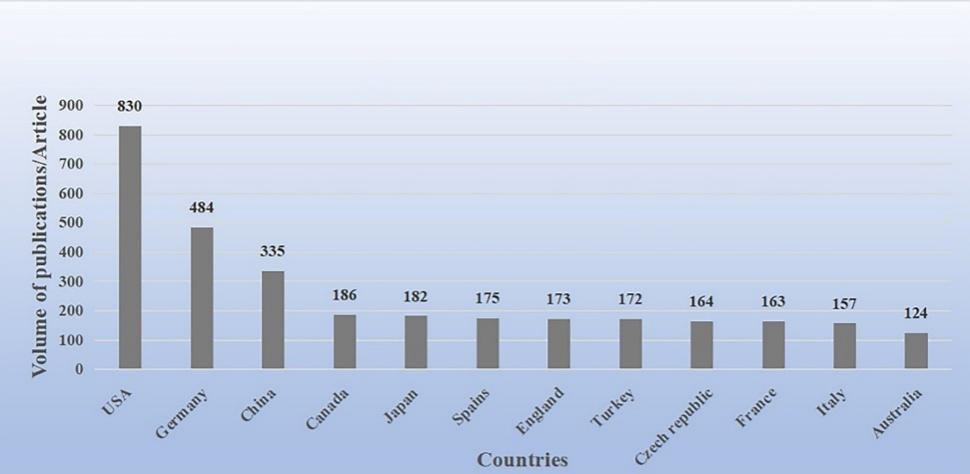


Supplementary Figure 1. Countries with more than 100 publications on Brassicaceae during 2002-2022.
